# Evaluation of nuclear NF-κB, transglutaminase2, and ERCC1 as predictors of platinum resistance in testicular tumors

**DOI:** 10.1590/S1677-5538.IBJU.2019.0011

**Published:** 2020-02-20

**Authors:** Alan A. Azambuja, Paula Engroff, Bruna T. Silva, Roberta C. S. Zorzetti, Fernanda B. Morrone

**Affiliations:** 1 Pontifícia Universidade Católica do Rio Grande do Sul Escola de Medicina PUCRS e Hospital Mãe de Deus Porto Alegre Brasil Programa de Pós-Graduação em Medicina e Ciências da Saúde, Escola de Medicina, Pontifícia Universidade Católica do Rio Grande do Sul, PUCRS e Hospital Mãe de Deus, Porto Alegre, Brasil; 2 Pontifícia Universidade Católica do Rio Grande do Sul Instituto de Geriatria e Gerontologia PUCRS Porto Alegre Brasil Instituto de Geriatria e Gerontologia, Pontifícia Universidade Católica do Rio Grande do Sul, PUCRS, Porto Alegre, Brasil; 3 Pontifícia Universidade Católica do Rio Grande do Sul Escola de Medicina PUCRS Porto Alegre Brasil Escola de Medicina, Pontifícia Universidade Católica do Rio Grande do Sul, PUCRS, Porto Alegre, Brasil; 4 Pontifícia Universidade Católica do Rio Grande do Sul Escola de Ciências da Saúde Escola de Medicina e Laboratório de Farmacologia Aplicada Porto Alegre Brasil Programa de Pós-Graduação em Medicina e Ciências da Saúde, Escola de Medicina e Laboratório de Farmacologia Aplicada, Escola de Ciências da Saúde, Pontifícia Universidade Católica do Rio Grande do Sul, Porto Alegre, Brasil

**Keywords:** transglutaminase 2 [Supplementary Concept], Testicular Neoplasms, Cisplatin

## Abstract

**Purpose:**

Testicular germ cells tumor (TGCT) are associated with a high cure rate and are treated with platinum-based chemotherapy. However, a group of testicular cancer patients may have a very unfavorable evolution and insensitivity to the main therapeutic agent chemotherapy (CT) cisplatin. The aim of this study was to evaluate the risk of recurrence and overall survival related to the expression of nuclear factor kappa-B (NF-κB), transglutaminase 2 (TG2) and excision repair cross-complementation group 1 (ERCC1) in patients with TGCT treated with platinum combinations.

**Patients and Methods:**

A retrospective study was performed with TGCT patients treated with platinum-based chemotherapy. Immunohistochemical analysis was performed and the expression was correlated with clinical and laboratory data.

**Results:**

Fifty patients were included, the mean age was 28.4 years (18 to 45), and 76% were non-seminoma. All patients were treated with standard cisplatin, etoposide and bleomycin or cisplatin, and etoposide. Patient’s analyzed immunodetection for NF-κB, TG2, and ERCC1 were positive in 76%, 54% and 42%, respectively. Multivariate analysis identified that positive expressions to ERCC1 and NF-κB are independent risk factors for higher recurrence TGCT after chemotherapy (RR 2.96 and 3.16, respectively). Patients with positive expression of ERCC1 presented a poor overall survival rate for 10-year follow (p=0.001).

**Conclusions:**

The expression of ERCC1 and NF-κB give a worse prognosis for relapse, and only ERCC1 had an influence on the overall survival of TGCT patients treated with platinum-based chemotherapy. These may represent markers that predict poor clinical outcome and response to cisplatin.

## INTRODUCTION

Testicular tumors account for 1% of all cancers in men. It is most frequent in men 15–35 years old and thus involves always a dramatic diagnosis (1). The very majority are testicular germ cell tumors (TGCT), where 50% are seminomas, 40% non-seminomas and the others are mixed tumors (1, 2). Even with the advent of new drugs in chemotherapy, cisplatin remains the treatment regimen with most curative potential for testicular cancer (3). Cisplatin cytotoxic activity results of interactions with DNA and the inability to repair DNA strand can lead to tumor cell apoptosis (3-5). In fact, adducts between platinum and DNA inhibit cellular processes, such as replication, transcription, translation and DNA repair (3). The decrease in cellular respiration can produce reactive oxygen species, resulting in lipid peroxidation (4). Furthermore, cisplatin binding to the mitochondrial DNA leads to decreased ATP and thus the decrease in ATPase activity and modification of the calcium content (4).

However, a group of testicular cancer patients may have a very unfavorable evolution and insensitivity to the main therapeutic agent chemotherapy (CT), cisplatin. Around 20-30% of the cases relapse and a second line of CT is necessary (4). Several mechanisms of cisplatin resistance have been proposed. Studies have linked the expression of excision repair cross-complementation group 1 (ERCC1) gene to chemoresistance as well as to poor survival in many types of cancer such as non-small-cell lung cancer, ovarian and gastric tumors (6-9). In TGCT cancer cell lines it has been reported an association of the cisplatin non-sensitivity with high levels of ERCC1, suggesting that this marker could be a potential mediator of response to cisplatin and a prognostic factor (10). Likewise, the overexpression of ERCC1 and XPF in TGCT was previously described during the progression of seminoma to non-seminoma (11). In addition, the transcription factor nuclear factor kappa-B (NF-κB) has been described to mediate cisplatin resistance. NF-κB is involved in many cellular functions, including the regulation of apoptosis and platinum-based chemotherapy resistance (12). Other studies demonstrate its role in tumorigenesis, CT resistance and a worse prognosis in bladder and head and neck cancer (12-14). Another marker is transglutaminase 2 (TG2), a trans-peptidase with a wide distribution in various tissues that plays an important role in malignancy progression by suppressing apoptosis (15). It is overexpressed in several neoplasms such as breast, ovaries, pancreas, and colon (16). TG2 is considered a prognostic marker in various cancers, due to its participation in promoting malignant cell mobility, invasion, metastasis, and chemoresistance, especially by platinum (17). Further mechanisms can be involved in platinum resistance such as decreased tumor blood flow, reduced platinum uptake, increased efflux, decreased binding, DNA repair, alteration of antiapoptotic factors and effects of various signaling pathways, among others (18).

A previous study showed that high expression of ERCC1 was associated with non-sensitivity to cisplatin-based CT in patients with non-seminomas TGCT (10), but little is known about other mechanisms involved in platinum resistance in testicular cancer. Therefore, the identification of other molecular markers to platinum-resistance is essential to a better treatment selection, avoiding unnecessary toxicity associated with platinum-based CT. In this study, we assessed the correlation of NF-κB, TG2 and ERCC1 expression with clinical outcomes in patients with TGCT treated with standard platinum combinations.

## PATIENTS AND METHODS

### Study design and data collection

A retrospective study was performed to evaluate tumor markers of cisplatin resistance in patients with testicular cancer receiving chemotherapy treatment. Eligible patients included male individuals (aged 18 years or above) with the confirmed diagnosis of testicular germ cell tumors. Seventy-six (76) cases of patients diagnosed with testicular cancer were evaluated in the Oncology Department-Hospital São Lucas/PUCRS in the period 2001 to 2011. Twenty-six 26 patients were excluded from the study due to the following reasons: lack of adherence to treatment or follow-up, incomplete data and loss of paraffin blocks. Histological indicative of TGCT was required to confirm the diagnosis. Data collection was retrospectively done through medical chart analysis of the cases treated. Patient’s characteristics and tumor markers alpha-fetoprotein (AFP), beta-hCG and lactate dehydrogenase (LDH) were collected. The measurement of the tumor markers was made usually after the first-month post orchiectomy. The cut-off points and patient’s stratification risk were evaluated according to the International Germ Cell Consensus Classification (IGCCCG) (19). This study was approved by the Institutional Ethics Committee of PUCRS (CEP number 0804398).

### End-points

The endpoints were the relapse/recurrence rate and overall survival (OS). Recurrence was defined when the progression of disease in computer tomography was confirmed. OS was calculated from the time of diagnosis to the date of death. The follow-up time for recurrence and OS was of 120 months for any cause of death.

### Immunohistochemistry

To determine the expression of ERCC1, NF-κB and TG2 (Santa Cruz Biotechnology, Inc., Santa Cruz, CA, EUA), in the germ cell tumors we performed immunohistochemistry assay ((9, 13, 20). The tumors were excised after surgery and fixed in buffered neutral formalin, sectioned, processed to paraffin wax and mounted onto a microscope slide. For the immunostaining study, sections were deparaffinized and rehydrated. The sections were submitted to antigenic retrieval being incubated with TRIS-EDTA, pH 9.0 for 30 minutes in a water bath at 98°C. For detection of the antibodies, it was used the REVEAL Biotin-Free Detection System (Spring Bioscience). The incubation of the primary antibodies (anti-NF-κB p65, clone C22B4, anti-TG2 clone CUB7402, anti-ERCC1, clone 8F1) was performed overnight at 4°C. Chromogenic detection (DAB) was used and the slides were counterstained with hematoxylin. The slides were mounted with glass coverslips using Canada Balsam and viewed with a microscope equipped with a camera. Images were captured in 400x amplification. For control experiments, primary antibody was omitted and evaluated for specificity or background staining levels. ERCC1, NF-κB and TG2 expression was considered as positive or negative by a pathologist blinded to the clinical outcome of each patient. The staining intensity was graded on a scale of 0 to 3. The percentage of positive nuclei was calculated for each specimen, and a proportion score was assigned (0 if 0%, 0.1 if 1 to 9%, 0.5 if 10 to 49% and 1.0 if 50% or more), as previously described (21). This proportion score was multiplied by the staining intensity of nuclei to obtain a final semi-quantitative H score. The median value of all the H scores was a priori chosen as a cut off point for separating positive tumors from negative tumors.

### Statistical analysis

Quantitative data were described as mean±standard deviation (SD). Categorical data were presented by counts and percentages. Fisher’s exact test or Pearson Chi-Square were used in categorical data. To obtain estimates of the association between ERCC1, NF-κB and TG2 markers with the occurrence of relapse we used a negative binomial regression model that provided relative risk estimates and their 95% confidence intervals. The relative risk was adjusted by age, stage, AFP, beta-hCG, and LDH histology. The differences in OS between categories of interest were analyzed using the log-rank test, and the hazard ratios (HRs) of the adjusted ERCC1, NF-κB and TG2 were calculated using the Cox model. The survival curves were constructed using the Kaplan-Meier method and significant between-group differences were assessed by the log-rank test. The significance level was set at α=0.05. Data were analyzed with the aid of the program SPSS version 22 and Sigma-Plot version 11.

## RESULTS

In this study, we assessed the correlation of NF-κB, TG2 and ERCC1 expression to clinical outcomes in 50 patients with TGCT treated with standard platinum combinations. The characteristics of the patients studied are presented in Table-1. Median age (range) of the group analyzed was 28.0 (18 to 45) years, 18 (36%) patients were clinical stage I, 10 (20%) were clinical stage II and 21 were stage III (42%), 12 cases (24%) were of seminoma and 38 cases (76%) of non-seminoma. Patient’s stratification risk for non-seminoma was 16% poor prognosis, 38% intermediate risk and 46% good prognosis and, 58% good prognosis and, 42% intermediate to the seminoma cases.

**Table 1:** Characteristics of testicular cancer patients studied (n=50).

		n	(%)
**Age (years)**			
	median	28.0	
	range	18 to 45	
**Clinical stage, no. (%)**			
	CS I	18	(36)
	CS II	10	(20)
	CS III	21	(42)
**Histology, no. (%)**			
	Seminoma	12	(24)
	Non-seminoma	38	(76)
**Risk stratification, no. (%)**			
	**Seminoma**		
	Good	7	(58)
	Intermediate	5	(42)
	**Non-seminoma**		
	Good	18	(46)
	Intermediate	14	(38)
	Poor	6	(16)
**Chemotherapy, no. (%)**			
	BEP (x3)	36	(72)
	EP (x4)	14	(28)
**Radiotherapy, no. (%)**		4	(8)
**Alpha-fetoprotein, no. (%)**			
	<1000ng/mL	16	(32)
	≥1000ng/mL	34	(68)
**Beta-hCG, no. (%)**			
	<1000ng/mL	15	(30)
	≥1000ng/mL	35	(70)
**LDH, no. (%)**			
	<1.5 x normal	22	(44)
	≥1.5 x normal	28	(56)
**Recurrence**		22	(44)

Date are presented as mean±standard deviation, median, and range of counts (percentage).

**LDH** = Lactate dehydrogenase; **BEP** = bleomycin, etoposide, and cisplatin; **EP** = etoposide and cisplatin; **CS** = clinical stage

**Figure 1:**
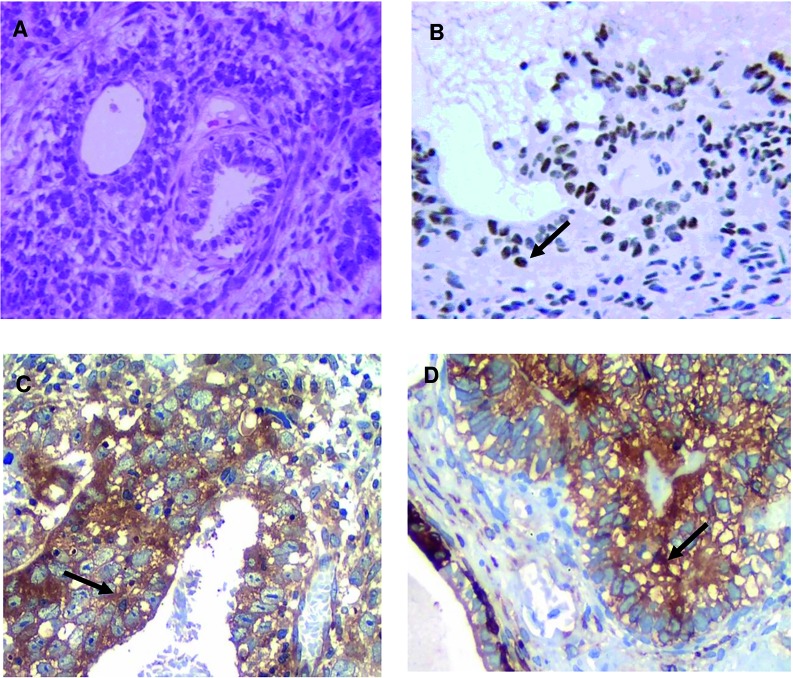
Representative images of H&E and immunostaining for the tumor markers (x400): A) H&E of non-seminoma testicular Tumor. Arrows indicate immunopositivity for: B) ERCC1; C) NF-κB; D) TG2.

The protocols of CT administered to the patients studied were in agreement with the currently first-line treatment pattern for TGCT (15). Patients received intravenously: BEP (cisplatin 20mg/m^2^ on days 1 to 5, etoposide 100mg/m2 on days 1 to 5 and bleomycin 30UI, on days 2, 9 and 16) or EP (cisplatin 20mg/m^2^, days 1 to 5 plus etoposide 100mg/m^2^ days 1 to 5), every 21 days, intravenously (22). In this study, 36 (72%) patients received BEP (x3), 14 (28%) received EP (x4), and 4 (8%) patients used radiotherapy after CT (Table-1). The assessment of tumor markers was made after the orchiectomy. The AFP, beta--hCG, and LDH were elevated, on the 30h day post orchiectomy, in 68%, 70% and 56% of cases, respectively (Table-1). Immunodetection for ERCC1, NF-κB, and TG2 markers was positive in 42%, 76% and 46% of the patient’s samples analyzed, respectively, and there were no statistically significant differences between seminoma and non-seminoma (Figures 1 A, B and C, Table-2).

**Table 2:** Immunohistochemical analysis of ERCC1, NF-κB, and TG2 in patients with testicular cancer (n=50).

Marker positive	Total no. (%)	Seminoma no. (%)	Non-seminoma no. (%)	P value
ERCC1	21 (42.0)	3 (14.3)	18 (85.7)	0.171
NF-κB	38 (76.0)	8 (21.1)	30 (78.9)	0.385
TG2	23 (46.0)	8 (34.8)	15 (65.2)	0.094

**ERCC1** = Excision repair cross-complementation group; **NF-κB** = nuclear factor kappa B; **TG2** = transglutaminase 2.

**Figure 2:**
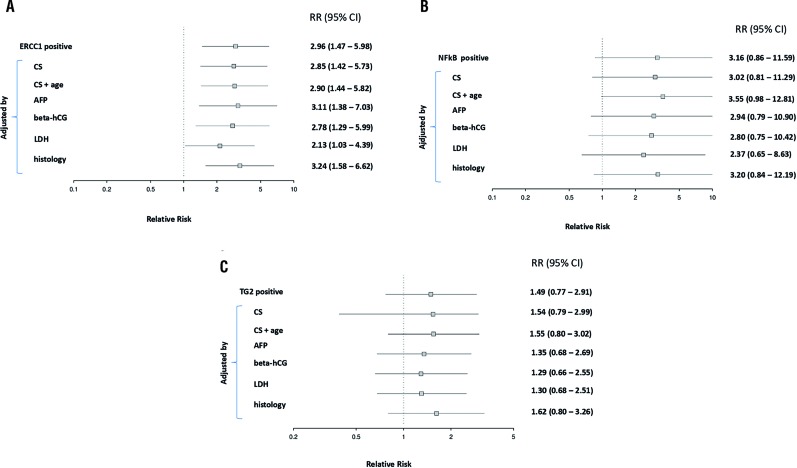
A) Relative risk for relapse to ERCC1 positive cases; stratified mode adjustments for factors such as age, clinical stage (CS), alpha-fetoprotein (AFP), beta-hCG, lactate dehydrogenase (LDH) and histology non-seminoma. B) The relative risk for relapse to NF-KB positive cases; stratified mode adjustments for factors such as age, clinical stage (CS), alphafetoprotein (AFP), beta-hCG, lactate dehydrogenase (LDH) and histology non-seminoma. C) The relative risk for relapse to TG2 positive cases; stratified mode adjustments for factors such as age, clinical stage (CS), alpha-fetoprotein (AFP), beta-hCG, lactate dehydrogenase (LDH) and histology non-seminoma. Amounts right corresponds to p and ranges (n=50).

The relative risk (RR) of ERCC1, NF-κB, and TG2 for testicular cancer relapse after completion of chemotherapy are depicted in Figure-2. It is presented also the adjusted RR for possible confounding factors in the outcome. The data on ERCC1 expression was significantly associated with a higher risk of relapse. When we adjusted stratified for factors such as age, clinical stage, alpha-fetoprotein, beta-hCG, lactate dehydrogenase, and histology non-seminoma, there was persistent the risk of recurrence (Figure-2A). Interestingly, we showed, for the first time, that the risk for relapse is around three times as high in the group NF-κB positive when compared to NF-κB negative, and this difference remains even after the adjustment of a potential factor of influence, with the exception of the tumor maker LDH (Figure-2B). No differences were observed with TG2 marker (Figure-2C). Interestingly, when we evaluated the impact of ERCC1 positive plus NF-kB positive versus ERCC1 positive plus NF-kB negative, there was a significant increase in the risk of recurrence for the markers combined positiveness (71.4% vs. 29.4%; p=0.001).

**Figure 3:**
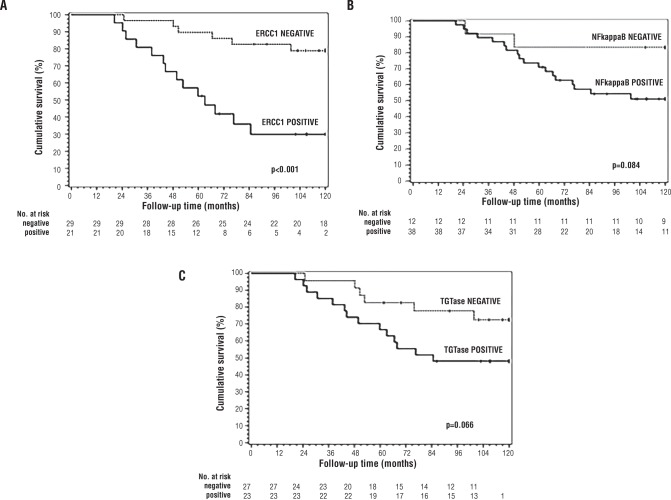
Kaplan-Meier estimate of overall survival probability according to ERCC1, p <0.001. A) NF-κB, p=0.084; B) and TG2, p=0.066; C). The differences in OS between categories of interest were analyzed using the log-rank test, and the significance level was set at α=0.05.

The evaluation of overall survival among patients with ERCC1-negative tumor, 1, 3, 5 and 10-year overall survival rate were 100%, 96%, 89% and 62%, compared to 100%, 85%, 57% and 9% for patients with positive expression of ERCC1 (p=0.001) (Figure-3A). The levels of NF-κB and TG2 protein expression had no significant influence on overall survival (Figures 3B and C).

## DISCUSSION

Platinum-based chemotherapy remains the first line of treatment of TGCT for more than 30 years (1). This study aimed to assess new molecular markers involved in cisplatin resistance, and their correlation with tumors relapse in patients with testicular tumors. In this study, the majority of patients studied were young males with TGCT non-seminoma and had a poor or intermediate prognosis classification. One of the main problems related to the recurrence of TGCT is platinum resistance and the mechanisms associated with cisplatin resistance involve many different cellular processes ((18, 23). Previous studies have demonstrated that platinum damage can be repaired by the nucleotide excision repair system, especially by ERCC1 (21). Our results showed that the expression of ERCC1 is associated with increased risk for TGCT relapse after treatment with platinum-based chemotherapy. When multivariate analysis was performed, none of the confounding factors in the outcome was able to change this point. These results show, in an important manner, that ERCC1 overexpression may predict that the curative chemotherapy has a relative risk of 2.96 to failure. In effect, predictive and prognostic values of ERCC1 expression have been studied in many solid tumors. It has been reported in some studies in ovarian, head and neck, and particularly in lung cancer that this marker could predict the response to chemotherapy (7, 9, 10, 24, 25). Corroborating to our results, Mendoza et al. (10) demonstrated that high levels of ERCC1 were associated with non-cisplatin sensitivity, suggesting that ERCC1 could be used as a potential indicator of the response to cisplatin and prognosis in non-sensitive TGCTs. Furthermore, the study of Olaussen et al. (9) demonstrated that the benefit of adjuvant chemotherapy with cisplatin was lost when there was a high expression of ERCC1 in the small-cell lung cancer tumor. In patients with squamous cell carcinoma, the expression of ERCC1 predicts a lower response to chemotherapy treatment (25). Interestingly, overexpression of ERCC1 gene seems to be associated with a reduction in the therapeutic efficacy of cisplatin, and the clinical response varies with polymorphisms ERCC1 (6). The mechanism by which ERCC1 contributes to cisplatin resistance involves a nucleoside excision repair, which removes platinum-DNA adducts and repairs the DNA double-strand breaks, and other reports mention an inherent biologic characteristic of the tumor (24, 25). Our results suggest that the evaluation of ERCC1 expression may contribute as a more accurate predictor of patient’s selection who are at increased risk of recurrence following standard treatment with cisplatin.

The data presented herein showed, for the first time, the expression of NF-κB in TGCT. The detection of high levels of NF-κB in patients with testicular cancer supports the hypothesis of a higher risk of recurrence after the treatment with cisplatin. Although there were no differences in the risk of recurrence for positive expression of NF-κB alone, we observed a significant increase in the risk of recurrence when we evaluated combined positive expression of ERCC1 plus NF-κB. The mechanism of resistance could be explained since cisplatin significantly increases NF-κB DNA binding activity, and NF-κB may antagonize apoptosis induced by cisplatin ((26, 27). It has been described that NF-κB inhibitors augment platinum activity against some cancer cell lines and tumor xenograft models ((28, 29). On the other hand, NF-κB activation increased cisplatin efficacy in some cell lines, and enhanced efficacy of higher cisplatin concentrations (30). For the other marker studied, there was no significant association between TG2 with the recurrence of TGCT.

It is worth to mention that due to its retrospective design, the number of clinical samples, and survival bias could potentially threaten the conclusions of this study. A further prospective cohort of patients should be performed in order to confirm the results achieved herein. Another limitation of this study is the inherent weaknesses of immunohistochemical staining, such as its semi-quantitative nature and interobserver variation. It is worth to consider that in this study the markers were analyzed only after surgery, which could limit the assessment.

In conclusion, we demonstrated that ERCC1 and NF-κB expression confer a worse prognosis for recurrence in patients with TGCT treated with standard platinum-based chemotherapy. The identification of resistance markers in TCGT patients who are potentially non-sensitive to cisplatin chemotherapy can improve their quality of life by avoiding the adverse effects caused by this agent.
